# Free fatty acids and cardiovascular outcome: a Chinese cohort study on stable coronary artery disease

**DOI:** 10.1186/s12986-017-0195-1

**Published:** 2017-06-26

**Authors:** Hui-Wen Zhang, Xi Zhao, Yuan-Lin Guo, Cheng-Gang Zhu, Na-Qiong Wu, Jing Sun, Geng Liu, Qian Dong, Jian-Jun Li

**Affiliations:** Division of Dyslipidemia, State Key Laboratory of Cardiovascular Disease, Fu Wai Hospital, National Center for Cardiovascular Diseases, Chinese Academy of Medical Sciences and Peking Union Medical College, No 167 BeiLiShi Road, XiCheng District, Beijing, 100037 China

**Keywords:** Free fatty acids, Cardiovascular events, Stable coronary artery disease

## Abstract

**Background:**

Studies have revealed that plasma level of free fatty acids (FFAs) is related to cardiovascular disease. However, the role of FFAs in clinical outcomes of stable coronary artery disease (SCAD) has been less evaluated. This study, therefore, was to investigate the predictive value of FFAs in Chinese cohort with angiography-proven SCAD.

**Methods:**

A total of 1670 consecutive patients with angina-like chest pain who underwent coronary angiography were enrolled and followed up for an average time of 36.9 months. The baseline characteristics were collected. Clinical and biochemical parameters of patients with or without cardiovascular events (CVEs) including all-cause death, non-fatal myocardial infarction, ischemic stroke and coronary revascularization were compared. According to the quartiles of baseline FFAs, patients were divided into four groups. The relationship of FFAs level and CVEs was evaluated.

**Results:**

During the follow-up period, 146 patients suffered from CVEs. Patients with CVEs had elevated plasma level of FFAs than patients without CVEs (*P* = 0.002). Using the Cox proportional hazards model, the unadjusted hazard ratio (HR) for CVEs in the fourth FFAs quartile was 2.09 (95% CI:1.32–3.33; *P* = 0.002) and was 1.80 (95% CI: 1.11–2.94; *P* = 0.018) after adjustment for traditional cardiovascular risk factors when compared with that in the first FFAs quartile.

**Conclusions:**

The present study on Chinese cohort with angiography-proven SCAD suggested that increased plasma level of FFAs was an independent risk factor of CVEs.

**Electronic supplementary material:**

The online version of this article (doi:10.1186/s12986-017-0195-1) contains supplementary material, which is available to authorized users.

## Background

Coronary artery disease (CAD) is the leading cause of death in worldwide because of its adverse clinical outcomes. Free fatty acids (FFAs), also named as non-esterified fatty acids, have attracted considerable attention due to their predictive value in CAD [1, 2]. FFAs are essential source of energy in human body originating from the adipose tissue, which provide 60%–70% of adenosine triphosphate to the heart cells. Interestingly, previous data have indicated that elevated plasma FFAs level can enhance oxidative stress, subsequently resulting in vascular endothelial dysfunction [3–6] and inflammation [7, 8]. Meanwhile, FFAs have been shown to influence the metabolize of lipids and glucose, which may aggravate the development of atherosclerosis and insulin resistance [9, 10]. Therefore, FFAs have been regarded as a risk marker of diabetes, obesity and cardiovascular diseases.

Although previous studies have suggested that plasma level of FFAs is related to the incidence and prognosis of cardiovascular disease [11–13], only two studies have revealed a positively predictive value of FFAs for clinical outcomes in patient with Stable CAD (SCAD) [12, 13]. In contrast, a study [14] on French cohort with male employees did not support the concept that FFAs is a marker for predicting the incidence of cardiovascular disease during a 15-year follow-up. The negative result was also found in the Quebec Cardiovascular Study cohort in participants free of cardiovascular disease [15]. Therefore, more researches are needed to determine whether FFAs are predictive for adverse cardiovascular events. In the present study, we enrolled a relatively large number of Chinese patients with SCAD to carefully examine the relationship between plasma level of FFAs and cardiovascular events (CVEs).

## Methods

### Study population

This protocol of the present study was complied with the Declaration of Helsinki, and was approved by the hospital ethics review board (Fu Wai Hospital and the National Center for Cardiovascular Diseases, Beijing, China). The informed consent was obtained from all the enrolled subjects.

From December 2011 through March 2013, a total of 2130 consecutive patients with angina-like chest pain referred for elective coronary angiography at our center were enrolled. SCAD was defined as described in our previous studies [16, 17], including patients with typical angina-like chest pain induced by exertion and/or positive treadmill exercise test, and had 50% diameter stenosis at least 1 of the 3 major coronary arteries or major branches assessed by elective coronary angiography. Exclusion criteria: acute coronary syndrome, heart failure (left ventricular ejection fraction, LVEF < 45%), severe liver and/or renal insufficiency, infectious or systematic inflammatory disease, thyroid dysfunction, malignant disease and patients with unavailable FFAs variables. Finally, a total of 1670 patients were included. The baseline, clinical and laboratory parameters were collected from all enrolled patients. Hypertension was diagnosed with repeated blood pressure measurements ≥140/90 mmHg or people who under anti-hypertensive drugs treatment. Type 2 diabetes was defined as fasting plasma glucose (FPG) ≥7.0 mmol/L and/or non-FPG ≥11.1 mmol/Lin multiple examinations or under treatment with insulin or oral hypoglycemic agents. Dyslipidemia was defined as the presence of fasting plasma total cholesterol (TC) ≥ 5.2 mmol/L (200 mg/dl) and/or triglyceride (TG) ≥ 1.7 mmol/L (150 mg/dl). Body mass index was calculated as BMI (kg/m2) = body weight (kg)/body height(m^2^).

### Laboratory analysis

Blood samples of all the enrolled patients were obtained from cubital vein after a 12-h overnight fasting and collected into EDTA-containing tubes. All samples were subsequently stored at −80 °C and analyzed immediately after thawing. The concentrations of plasma TC, TG, high-density lipoprotein cholesterol (HDL-C), high-density lipoprotein cholesterol (LDL-C), and FFAs were measured using an automatic biochemistry analyzer (Hitachi 7150, Tokyo, Japan). The concentrations of high-sensitive C-reactive protein (hs-CRP) were measured by immunoturbidometry (Beckmann Assay 360; Bera, CA, USA). Plasma hemoglobin A1c (HbA1c) levels were measured using the Tosoh G7 Automate HPLC Analyzer (TOSOH Bioscience, Japan).

### Definition of Events and Follow-up

The predefined endpoint outcomes of the present study including all-cause mortality, non-fatal myocardial infarction (MI), ischemic stroke and coronary revascularization (CRV) including percutaneous coronary intervention (PCI) or coronary artery bypass graft surgery (CABG). Follow-up data of the enrolled patients were collected by the trained nurses who were blinded to the aim of this study. We performed standardized telephone interviews at 12,24, and 36 months. The follow-up time of each patient was calculated as the number of months from the enrollment till the last traceable hospital outpatient or inpatient record or telephone interview before March 2016, and was censored on the date of the endpoint events occurred.

### Statistical analysis

Continuous variables were presented as mean ± standard deviation (SD) or median with ranges and categorical variables as percentages (%). Comparisons of continuous data between groups were performed with Student t test or ANOVA as appropriate, and χ^2^ test Fisher’s exact test were used for categorical data. The association of FFAs levels with events was described with calculated hazard ratios (HRs) and 95% confidence intervals (CIs) using Cox proportional hazard models. Cumulative survival and event-free survival rates were estimated via the Kaplan-Meier method and compared using a log-rank test. Correlations between FFAs and other established cardiovascular risks variables were examined using Spearman or Pearson correlation coefficient when appropriate. A *P* < 0.05 for two sided was considered as statistical significance. This statistical analysis was performed with SPSS 21.0 software (Chicago, IL).

## Results

After an average follow-up time of 36.9 months, a total of 1506 patients finally completed the follow-up visit and 146 (9.7%) patients suffered from CVEs, including all-cause death (*n* = 24), non-fatal MI (*n* = 20), ischemic stroke (*n* = 27), and CRV (*n* = 75). We compared the demographic characteristics between patients with CVEs and patients without CVEs as shown in Table 1. Data showed that patients with CVEs had a higher plasma level of FFAs than patients without CVEs (*P* < 0.05). Moreover, the group with CVEs presented a higher prevalence of hypertension, type 2 diabetes, and elevated level of FPG and HbA1c compared with that in patients without CVEs (*P* < 0.05, respectively). The mean age of the including subjects was 58.8 years old (range from 19 to 81 years old), and 73% of the enrolled participants were male patients. Given the wide age range from 19 to 81 years old of the participants, we compared the baseline characteristics according to age tertiles (the detailed characteristics were shown as Additional file 1: Table S1). Data showed no significant difference in the incidence of CVEs between age groups (tertile 1: *n* = 50,8.9% vs tertile 2: *n* = 48,9.6% vs tertile 3: *n* = 48,10.7%; *P* = 0.64). The mean value of FFAs was 0.43 ± 0.19 mmol/L (ranged from 0.03 to 1.40 mmol/L) in the enrolled population. According to the quartiles of plasma level of FFAs, the 1506 patients were divided into four groups, quartile 1 (≤0.3 mmol/L, *n* = 403), quartile 2 (0.31–0.40 mmol/L, *n* = 364), quartile 3 (0.41–0.53 mmol/L, *n* = 391), quartile 4 (>0.53 mmol/L, *n* = 348). The baseline characteristics and laboratory parameters of the included subjects according to FFAs quartiles were shown in Table 2. The group in the fourth quartile of FFAs appeared to have higher levels of TG, TC, BMI, FPG, HbA1c, hs-CRP and lower level of HDL-C (*P* < 0.05, respectively) when compared with the first quartile of FFAs. Patients in the fourth quartile of FFAs also showed higher prevalence of type 2 diabetes, dyslipidemia and hypertension (*P* < 0.05, respectively) when compared with the first quartile of FFAs. There was no significant difference in proportion of lipid-lowering drugs treatment (*P* > 0.05) among the four groups. There was a significantly increased incidence of CVEs with the elevation of plasma FFAs level (quartile 1: *n* = 28, 6.9% vs quartile 2: *n* = 31, 8.5% vs quartile 3: *n* = 38, 9.7% vs quartile 4: *n* = 49, 14.1%; *P* = 0.008).

**Table 1:** Demographic characteristics in patients with or without cardiovascular events (CVEs)

Variables	Total (*n* = 1506)	Patients with CVEs (*n* = 146)	Patients without CVEs (*n* = 1360)	*P* value
Baseline characteristics				
Age (years)	58.76 ± 10.09	58.65 ± 10.04	59.90 ± 10.16	0.23
Male (n,%)	1099(73%)	100(68.5%)	999(73.5%)	0.20
Family history of CAD (n,%)	204(13.5)	22(15.1)	182(13.4)	0.57
Hypertension (n,%)	971(64.5%)	108(74%)	863(63.5%)	0.01
Dyslipidemia (n,%)	1165(77.4%)	114(78.1%)	1051(77.3%)	0.83
Diabetes (n,%)	431(28.6%)	55(37.3%)	376(27.6%)	0.01
Current smoking (n,%)	786(52.2%)	66(45.2%)	720(52.9%)	0.08
BMI (kg/m^2^)	25.57 ± 3.16	25.58 ± 3.18	25.51 ± 2.96	0.80
Lipid-lowering treatment (n,%)	630(41.8%)	54(37.0%)	576(42.4%)	0.21
Aspirin treatment (n,%)	1480(98.3%)	143(97.9%)	1337(98.3%)	0.75
Laboratory parameters				
TG (mmol/L)	1.73 ± 1.00	1.78 ± 1.17	1.73 ± 0.98	0.54
TC (mmol/L)	4.14 ± 1.13	4.25 ± 1.13	4.13 ± 1.13	0.22
LDL-C (mmol/L)	2.45 ± 0.89	2.53 ± 0.95	2.44 ± 0.88	0.27
HDL-C (mmol/L)	1.08 ± 0.26	1.08 ± 0.28	1.08 ± 0.26	0.88
FFAs (mmol/L)	0.43 ± 0.19	0.47 ± 0.22	0.42 ± 0.19	0.002
HbA1c (%)	6.41 ± 1.14	6.64 ± 1.33	6.39 ± 1.12	0.03
FPG (mmol/L)	5.62 ± 1.60	5.92 ± 1.85	5.59 ± 1.57	0.04
LVEF(%)	62.53 ± 8.61	61.81 ± 9.03	62.72 ± 8.54	0.07
NT-pro-BNP (fmol/ml)	728.27 ± 532.25	763.55 ± 589.38	713.71 ± 494.94	0.09
Creatine (umol/L)	74.77 ± 15.20	76.35 ± 15.98	74.50 ± 15.09	0.12
hs-CRP (mg/L)	2.88(0.01–17.69)	3.04(0.01–13.73)	2.87(0.01–17.69)	0.12
cTnI (ng/ml)	0.07(0.001–7.37)	0.08(0.001–2.91)	0.07(0.001–7.37)	0.28

Values are expressed as mean ± SD, median with range, or n (%). SD: Standard deviation

*CAD* coronary artery disease, *BMI* body mass index, *TC* total cholesterol, *TG* triglyceride, *LDL-C* low-density lipoprotein cholesterol, *HDL-C* high-density lipoprotein cholesterol, *HbA1c* hemoglobin A1c, *FPG* fasting plasma glucose, *LVEF* left ventricular ejection fraction, *NT-pro-BNP* N-terminal–pro-brain natriuretic peptide, *hs-CRP* high sensitivity C-reactive protein, *cTnI* cardiac troponin I

**Table 2:** Demographic characteristics stratified by FFAs quartiles (mmol/L)

Variables	Quartile 1 (≤0.30) (*n* = 403)	Quartile 2 (0.31–0.40) (*n* = 364)	Quartile 3 (0.41–0.53) (*n* = 391)	Quartile 4 (>0.53) (*n* = 348)	*P* Value
Baseline characteristics					
Age (years)	58.65 ± 9.70	58.14 ± 10.15	58.50 ± 10.31	59.90 ± 10.16	0.15
Male (n,%)	292(72.5%)	283(77.7%)	295(75.4%)	229(65.8%)	0.002
Family history of CAD (n,%)	49(12.2%)	45(12.4%)	62(16.0%)	48(13.8%)	0.41
Hypertension (n,%)	226(56.5%)	228(63.7%)	269(69.0%)	248(71.3%)	<0.001
Dyslipidemia (n,%)	293(73.3%)	267(74.8%)	315(80.6%)	290(83.3%)	0.001
Diabetes (n,%)	72(18%)	77(21.6%)	127(32.5%)	115(33%)	<0.001
Current smoking (n,%)	220(55%)	193(53.6%)	208(53.3%)	165(47.6%)	0.22
BMI (kg/m^2^)	25.00 ± 3.24	25.54 ± 2.96	25.76 ± 3.19	26.02 ± 3.13	<0.001
Lipid-lowering treatment (n,%)	170(42.2%)	154(42.3%)	167(42.7%)	139(39.9%)	0.88
Aspirin treatment (n,%)	402(99.8%)	354(97.3%)	385(98.5%)	339(97.4%)	0.03
Laboratory parameters					
TG (mmol/L)	1.48 ± 0.60	1.75 ± 1.00	1.84 ± 1.02	1.91 ± 1.23	<0.001
TC (mmol/L)	4.03 ± 1.00	4.03 ± 0.94	4.17 ± 1.06	4.36 ± 1.47	0.002
LDL-C (mmol/L)	2.43 ± 0.88	2.36 ± 0.80	2.47 ± 0.90	2.55 ± 0.96	0.07
HDL-C (mmol/L)	1.08 ± 0.26	1.05 ± 0.23	1.07 ± 0.26	1.12 ± 0.28	0.02
FFAs (mmol/L)	0.23 ± 0.06	0.35 ± 0.03	0.46 ± 0.04	0.69 ± 0.17	<0.001
HbA1c (%)	6.17 ± 0.89	6.19 ± 1.03	6.55 ± 1.20	6.77 ± 1.32	<0.001
FPG (mmol/L)	5.22 ± 1.13	5.33 ± 1.31	5.75 ± 1.62	6.21 ± 2.06	<0.001
LVEF(%)	62.53 ± 8.94	62.59 ± 8.83	62.79 ± 8.12	62.18 ± 8.57	0.82
NT-pro-BNP (fmol/ml)	712.62 ± 463.92	691.97 ± 409.19	709.71 ± 516.94	805.07 ± 703.82	0.07
Creatine (umol/L)	74.17 ± 14.65	75.42 ± 14.07	75.04 ± 14.73	74.49 ± 17.35	0.30
hs-CRP (mg/L)	2.31(0.01–15.15)	2.61(0.01–16.51)	3.42(0.01–17.17)	3.22(0.01–17.69)	<0.001
cTnI (ng/ml)	0.08(0.001–6.03)	0.06(0.001–6.01)	0.09(0.001–7.37)	0.07(0.001–4.46)	0.57
Cardiovascular events (n,%)	28(6.9%)	31(8.5%)	38(9.7%)	49(14.1%)	0.008

Values are expressed as mean ± SD, median with range, or n (%). SD: Standard deviation

*CAD* coronary artery disease, *BMI* body mass index, *TC* total cholesterol, *TG* triglyceride, *LDL-C* low-density lipoprotein cholesterol, *HDL-C* high-density lipoprotein cholesterol, *HbA1c* hemoglobin A1c, *FPG* fasting plasma glucose, *LVEF* left ventricular ejection fraction, *NT-pro-BNP* N-terminal–pro-brain natriuretic peptide, *hs-CRP* high sensitivity C-reactive protein, *cTnI* cardiac troponin I

Data from our study showed a strong association between baseline plasma level of FFAs and incidence of all endpoint events as established in Table 3. The unadjusted HR for CVEs in the fourth FFAs quartile was 2.09 (95% CI 1.32–3.33; *P* = 0.002) when compared with subjects in the first FFA quartile. The multivariate analysis showed that plasma level of FFAs was an independent predictor for CVEs after adjusting for age, gender (HR:2.06, 95% CI:1.29–3.27; *P* = 0.002). FFAs level remained to be an independent predictor of adverse outcomes after adjusted for potential confounders including hypertension, diabetes, BMI, current smoking, and family history of CAD (HR:1.82, 95% CI:1.13–2.93; *P* = 0.014). Further adjusted for HbA1c, TC, TG, HDL-C, hs-CRP and cardiac troponin I. the result remained significant (HR: 1.80, 95% CI: 1.11–2.94; *P* = 0.018). In the study group, the incidence of CVEs in the fourth FFAs quartile was 4.96 per 100 person years compared to 2.46 per 100 person years in the first quartile (*P* < 0.001). As shown in Table 4, we additionally evaluated the relationship between plasma FFAs level and all-cause death, non-fatal MI, stroke and CRV respectively. Data indicated that FFAs level was also an independent risk factor of all-cause death (adjusted HR:4.11, 95% CI:1.23–13.73; *P* = 0.02), while there was no significant association between FFAs and non-fatal MI (adjusted HR:1.71, 95% CI:0.40–7.39; *P* = 0.47), stroke (adjusted HR:1.24, 95% CI:0.43–7.55; *P* = 0.68) in the present study. The unadjusted HR of fourth quartile of FFAs (first quartile as reference) in predicting for CRV was 1.92 (95% CI:1.02–3.93; *P* = 0.03), however, this association disappeared when further adjusted for conventional risk factors (adjusted HR:1.65, 95% CI:0.82–3.33; *P* = 0.16).

**Table 3:** Risk of cardiovascular events based on the quartiles of FFAs (first quartile as reference)

	Quartile 1 (≤0.30,*n* = 403)	Quartile 2 (0.31–0.40,*n* = 364)	Quartile 3 (0.41–0.53,*n* = 391)	Quartile 4 (>0.53,*n* = 348)
Incident cases	28	31	38	49
*P* value	-	0.63	0.16	0.001
Person-years	1138.92	1024.78	1138.51	987.50
Incidence/100 person-years	2.46	3.03	3.34	4.96
*P* value	-	0.25	0.03	<0.001
Univariate	1.00 (Ref.)	1.24(0.74–2.06)	1.42 (0.87–2.32)	2.09(1.32–3.33)
*P* value	-	0.41	0.16	0.002
Model 1^a^	1.00 (Ref.)	1.26(0.75–2.09)	1.15 (0.88–2.33)	2.06 (1.29–3.27)
*P* value	-	0.38	0.15	0.002
Model 2^b^	1.00 (Ref.)	1.20(0.72–2.01)	1.29(0.79–2.13)	1.82(1.13–2.93)
*P* value	-	0.49	0.31	0.014
Model 3^c^	1.00 (Ref.)	1.16(0.69–1.96)	1.30(0.79–2.15)	1.80 (1.11–2.94)
*P* value	-	0.58	0.31	0.018

*HRs* Hazard ratios, *CI* Confidence interval

^a^Adjusted for age and sex

^b^Additionally adjusted for hypertension, diabetes, body mass index, current smoking, and family history of coronary artery disease

^c^Additionally adjusted for hemoglobinA1c, total cholesterol, triglycerides, high-density lipoprotein cholesterol, high sensitivity C-reactive protein, and cardiac Troponin I

**Table 4:** Univariate and multivariate Cox hazard for different endpoint based on the fourth quartile of FFAs (first quartile as reference)

	Univariate		Multivariate	
	HRs (95% CIs)	*P* value	HRs (95% CIs)	*P* value
All-cause death	3.55(1.14–10.99)	0.03	4.11(1.23–13.73)	0.02
No-fatal MI	2.22(0.59–9.50)	0.22	1.71 (0.40–7.39)	0.47
Stroke	1.71 (0.40–7.39)	0.40	1.24(0.43–7.55)	0.68
CRV	1.92 (1.02–3.93)	0.03	1.65 (0.82–3.33)	0.16

*HRs* Hazard ratios, *CI* Confidence interval, *MI* Myocardial infarction, *CRV* Coronary revascularization

Multivariate model adjusted for age, sex, hypertension, diabetes, body mass index, current smoking, and family history of coronary artery disease, hemoglobinA1c, total cholesterol, triglycerides, high-density lipoprotein cholesterol, high sensitivity C-reactive protein and cardiac Troponin I

**Fig. 1:**
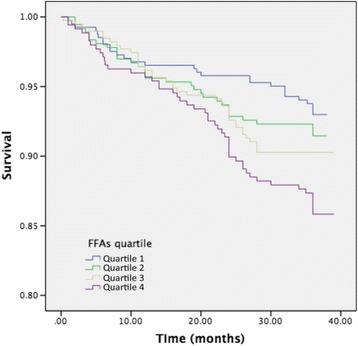
The Kaplan-Meier curves for cumulative event-free survival based on the quartiles of baseline FFAs levels

The Kaplan-Meier curves for cumulative event-free survival based on the quartiles of baseline FFAs levels were presented in Fig. 1, the highest quartile of plasma FFAs was positively associated with increase of CVEs when compared with the lowest quartile. We further investigated the relation between FFAs with established and emerging risk factors of cardiovascular disease. For the Spearman correlations analysis, furthermore, plasma level of FFAs was positively correlated with FPG (*r* = 0.239, *P* < 0.001), HbA1c (*r* = 0.215, *P* < 0.001), TC (*r* = 0.149, *P* < 0.001) and hs-CRP (*r* = 0.127, *P* < 0.001).

## Discussion

Data from our relatively large prospective Chinese cohort mainly revealed that plasma level of FFAs was an independent risk maker for evaluating future CVEs predefined as all-cause mortality, MI, ischemic stroke and CRV. Our data suggested that higher FFAs level predicted for a worse outcome in patients with angiography-proven SCAD and the observed positive association of FFAs with CVEs did not alter even after adjustment of conventional cardiovascular risk factors.

Although FFAs have been uncovered for many years, the investigations of their value in clinical diagnosis and prognosis of metabolic disorders are still of great interests. FFAs were mainly released from the storage in adipose tissue, which were originally considered to be of great importance to supply physiological source of energy for human body [2]. The physiological functions of FFAs included cell membrane formation, cellular signal transduction and regulate glucose metabolism [3, 4]. Therefore, several potential mechanisms may account for the findings in the present study. On the one hand, FFAs induced oxidative stress and inflammatory reaction [5, 6], leading to an acceleration in the rupture of atherosclerotic lesions and the progress atherosclerosis in patients with CAD. It was also reported that FFAs could induce inflammation and injury vascular endothelial cells [7, 8], which was essential etiology in the incidence and development of artery atherosclerosis [18–21]. In addition, elevated plasma level of FFAs could influence glucose production [9], which was demonstrated to be a risk marker for insulin resistant. In the previous studies, FFAs were revealed to be associated with metabolic syndrome as obesity and type 2 diabetes [22–24]. In our study, we also found positive association between FFAs and FPG (*r* = 0.239, *P* < 0.05), HbA1c (*r* = 0.215, *P* < 0.05), which was in consistent with previous studies [22–24]. Moreover, high level of FFAs was also found to play a role in arrhythmia [25, 26] and myocardial dysfunction [27–29]. Thereby, studies on the role of FFAs in predicting for atherosclerotic cardiovascular disease are benefit for improving strategies for prevention and therapy in CAD.

Several studies have investigated the relationship between FFAs and CAD previously, the conclusions were still in controversial. More recently, a prospective study of 3300 white individuals reported by Pliz et al. [12] have revealed that FFAs are independently associated with cardiac mortality in patients with angiographic CAD (HR:1.94, 95% CI:1.32–2.85; *P* = 0.001), stable CAD (HR:1.88, 95% CI:1.19–2.96; *P* = 0.007) and unstable CAD (HR:2.22, 95% CI:1.03–4.77; *P* = 0.041). Furthermore, Pliz [30] have also suggested that elevated level of FFAs is an independent risk factor for future sudden cardiac death (HR:1.76, 95% CI:1.03–3.00; *P* = 0.038) in 2231 angiographic CAD patients after 6.85-year follow-up. In addition, data from Breitling [13] included 1206 participants with 8 years of follow-up revealed an increased mortality associated with high levels of FFAs in SCAD (HR:2.071, 95% CI:1.066–4.021; *P* = 0.032). Michael et al. [31] showed that elevated plasma concentrations of FFAs were associated with cardiovascular and non-cardiovascular mortality in 4707 old subjects (≥65 years) from The Cardiovascular Health Study. Conversely, another prospective study [14] showed no significant association between FFAs and CVD mortality after adjustment for traditional risk factors in 4589 middle-aged men (mean age 48.8 years old). This result could be attributed to that plasma concentration of FFAs was influenced by factors such as stress, nutritional and smoking status of the enrolled patients since all the participants were men. Another hypothesis was that the metabolism of FFAs which could influence the contribution the cardiovascular risk might vary among different individuals [14]. A negative result was also found in a prospective research of Quebec Cardiovascular Study cohort [15] which suggested that elevated plasma FFAs concentrations were not associated with an increased risk of ischemia heart disease, it could be due to the fact that participants had normal range of plasma level of FFAs and the relatively small sample size of this study. Apparently, more researches are needed for performing FFAs-related outcomes. However, data regarding the role of FFAs in predicting cardiovascular outcome in Chinese patients with SCAD are not available. Here, in the present study, we conducted the relatively large prospective cohort in Chinese patients with SCAD, data showed that plasma level of FFAs was higher in patients with CVEs compared with patients without CVEs, and a significantly elevated incidence of CVEs was observed as the level of FFAs increased. The present study validated that FFAs were independently associated with CVEs even adjusted for established risk factors such as gender, current smoking, hypertension, diabetes and lipids level (adjusted HR 1.80, 95% CI: 1.11–2.94; *P* = 0.018) in patients with SCAD among Chinese cohort. We also revealed a significant value of FFAs in predicting for all-cause death (adjusted HR:4.11, 95% CI:1.23–13.73; *P* = 0.022). Our study, obviously, provided novel information with regard to the role of FFAs in SCAD. In addition, measurement of plasma FFAs levels is convenient and at less costs. Therefore, data from our study provided important clue to make more effective strategies for secondary prevention and therapy in patients with SCAD.

There were several limitations in the present study. Patients from a single-center study was the first limitation which may introduce a potential selection bias. Secondly, we have only measured the plasma level of FFAs for a single examination, since plasma concentration of FFAs could be influenced by the nutritional state, stress, physical activity and period of disease. And the change of FFAs level over the development of SCAD was not clear. Therefore, once measurement of fasting FFA may not accurately reflect the variations of FFAs in circulation. Then, the follow-up time of this study was relatively short. Therefore, further investigations with larger population and longer follow-up time are needed to confirm our study.

## Conclusions

In summary, data from our study validated that plasma level of FFAs was an independent risk factor for predicting CVEs in Chinese patients with SCAD. Elevated FFAs levels were related to a worse prognosis in these subjects. Our study suggested the potential of FFA-target strategies may improve the prognosis in patients with SCAD. Further studies are needed to confirm our study.

## Additional file

Additional file 1: Table S1. Demographic characteristics stratified by age tertiles (years). (DOCX 104 kb)

## Abbreviations

95% CIs: 95% confidence intervals
BMI: Body mass index
CABG: Coronary artery bypass graft surgery
CRV: Coronary revascularization
cTnI: cardiac troponin I
CVEs: Cardiovascular events
FFAs: Free fatty acids
FPG: Fasting plasma glucose
HbA1c: Hemoglobin A1c
HDL-C: High-density lipoprotein cholesterol
HRs: Hazard ratios
hs-CRP: High sensitivity C-reactive protein
LDL-C: Low-density lipoprotein cholesterol
MI: Myocardial infarction
PCI: Percutaneous coronary intervention
SCAD: Stable coronary artery disease
TC: Total cholesterol
TG: Triglyceride
