# Supplementary material for: Free fatty acids and cardiovascular outcome: a Chinese cohort study on stable coronary artery disease
Source: Nutr Metab (Lond). 2017 Jun 26;14:41. doi: 10.1186/s12986-017-0195-1 (PMC5485743; doi:10.1186/s12986-017-0195-1)
Supplement: Additional file 1: Table S1. — Demographic characteristics stratified by age tertiles (years). (DOCX 104 kb) [file 12986_2017_195_MOESM1_ESM.docx]

**Table S1.** Demographic characteristics stratified by age tertiles (years).

| Variables | Tertile 1  (≤55)  (n=558) | Tertile 2  (56-65)  (n=501) | Tertile 3  (>65)  (n=447) | | P Value |  |  |
| --- | --- | --- | --- | --- | --- | --- | --- |
| Baseline  characteristics |  |  |  | |  |  |  |
| Male (%) | 470(84.2%) | 338(67.5%) | 291(65.1%) | | <0.001 |  |  |
| Family history of  CAD (n,%) | 97(17.4%) | 71(14.2%) | 36(8.1%) | | <0.001 |  |  |
| Hypertension (n,%) | 327(58.6%) | 319(63.7%) | 325(72.7%) | | <0.001 |  |  |
| Dyslipidemia (n,%) | 424(76%) | 392(78.2%) | 349(78.8%) | | 0.62 |  |  |
| Diabetes (n,%) | 145(26%) | 147(29.3%) | 139(31.1%) | | 0.19 |  |  |
| Current smoking (n,%) | 362(64.9%) | 241(48.1%) | 183(40.9%) | | <0.001 |  |  |
| BMI (kg/m^2^) | 26.21±3.16 | 25.56±3.13 | 24.78±3.00 | | <0.001 |  |  |
| Statins treatment (n,%) | 247(44.3%) | 202(40.3%) | 181(40.5%) | | 0.34 |  |  |
| Aspirin treatment (n,%) | | 549(98.4%) | 492(98.2%) | 439(98.2%) | 0.97 | | |
| Laboratory  parameters |  |  |  | |  |  |  |
| TG (mmol/L) | 1.86±1.00 | 1.81±1.18 | 1.50±0.70 | | <0.001 |  |  |
| TC (mmol/L) | 4.07±1.25 | 4.26±1.13 | 4.10±0.97 | | 0.67 |  |  |
| LDL-C (mmol/L) | 2.40±0.90 | 2.53±0.93 | 2.44±0.81 | | 0.47 |  |  |
| HDL-C (mmol/L) | 1.01±0.24 | 1.09±0.26 | 1.15±0.27 | | <0.001 |  |  |
| FFAs (mmol/L) | 0.41±0.18 | 0.43±0.19 | 0.44±0.21 | | 0.06 |  |  |
| HbA1c (%) | 6.32±1.17 | 6.44±1.14 | 6.50±1.10 | | 0.01 |  |  |
| FPG (mmol/L) | 5.60±1.66 | 5.61±1.56 | 5.65±1.57 | | 0.85 |  |  |
| LVEF(%) | 62.56±7.45 | 62.19±9.62 | 62.86±8.77 | | 0.55 |  |  |
| NT-pro-BNP (fmol/ml) | 627.80±353.63 | 731.71±542.08 | 850.32±665.91 | | <0.001 |  |  |
| Creatine (umol/L) | 73.69±13.47 | 73.57±14.61 | 77.48±17.41 | | <0.001 |  |  |
| hs-CRP (mg/L) | 2.70(0.01-17.69) | 3.04(0.01-17.17) | 2.93(0.01-15.82) | | 0.30 |  |  |
| cTnI (ng/ml) | 0.07(0.001-4.03) | 0.07(0.001-7.37) | 0.09(0.001-6.04) | | 0.51 |  |  |
| Cardivascular events (n,%) | 50(8.9%) | 48(9.6%) | 48(10.7%) | | 0.64 |  |  |

Values are expressed as mean±SD, median with range, or n (%). SD: Standard deviation.

CAD: coronary artery disease; BMI: body mass index; TC: total cholesterol; TG: triglyceride; LDL-C: low-density lipoprotein cholesterol; HDL-C: high-density lipoprotein cholesterol; HbA1c: hemoglobin A1c; FPG: fasting plasma glucose; LVEF: left ventricular ejection fraction; NT-pro-BNP: N-terminal–pro-brain natriuretic peptide; hs-CRP: high sensitivity C-reactive protein; cTnI: cardiac troponin I.
